# Is There a Future for Non-invasive Brain Stimulation as a Therapeutic Tool?

**DOI:** 10.3389/fneur.2018.01146

**Published:** 2019-01-24

**Authors:** Carmen Terranova, Vincenzo Rizzo, Alberto Cacciola, Gaetana Chillemi, Alessandro Calamuneri, Demetrio Milardi, Angelo Quartarone

**Affiliations:** ^1^Department of Clinical and Experimental Medicine, University of Messina, Messina, Italy; ^2^Department of Biomedical, Dental Sciences and Morphological and Functional Images, University of Messina, Messina, Italy; ^3^IRCCS Centro Neurolesi ‘Bonino Pulejo’, Messina, Italy

**Keywords:** neuroplasticity, rTMS, tDCS, NIBS, neuropsychiatric disorders

## Abstract

Several techniques and protocols of non-invasive transcranial brain stimulation (NIBS), including transcranial magnetic and electrical stimuli, have been developed in the past decades. These techniques can induce long lasting changes in cortical excitability by promoting synaptic plasticity and thus may represent a therapeutic option in neuropsychiatric disorders. On the other hand, despite these techniques have become popular, the fragility and variability of the after effects are the major challenges that non-invasive transcranial brain stimulation currentlyfaces. Several factors may account for such a variability such as biological variations, measurement reproducibility, and the neuronal state of the stimulated area. One possible strategy, to reduce this variability is to monitor the neuronal state in real time using EEG and trigger TMS pulses only at pre-defined state. In addition, another strategy under study is to use the spaced application of multiple NIBS protocols within a session to improve the reliability and extend the duration of NIBS effects. Further studies, although time consuming, are required for improving the so far limited effect sizes of NIBS protocols for treatment of neurological or psychiatric disorders.

## Introduction

Transcranial magnetic stimulation (TMS) and transcranial direct current stimulation (tDCS) are the most popular techniques of non-invasive transcranial brain stimulation (NIBS).

TMS was introduced in the clinical use in 1985 as a tool to investigate the integrity and the function of human cortico-spinal system (1). Motor evoked potentials can be easily obtained and measured from the contralateral muscles of the stimulated hemisphere; the reproducibility of the responses allowed TMS to become a standard tool in clinical neurophysiology.

The magnetic field produced by TMS easily penetrates the scalp and the skull inducing an electric field in the area just beneath the coil in a painless way (2). The induced electrical field activates the axons of the neurons in the cortex and sub-cortical white better rather than then cell bodies, which have a higher threshold.

TMS produces local effects immediately under the coil and/or remote effects activating axons to or from the site of stimulation. The outcome of such stimulation is quite complex, resulting from a combination of excitatory, and inhibitory effects, that is far away from the organized patterns of activity that occur in natural behaviors.

An alternative way of modulating cortical excitability is to apply a weak current (1–2 mA) to the brain for 5–20 min using a pair of saline-sponged electrodes, one placed over the target cortical area and the other at distance (3). A significant modulation of cortical excitability can be obtained by changing the polarity of the current. Anodal tDCS depolarizes neurons, raising cortical excitability, while cathodal tDCS hyperpolarizes neurons reducing excitability (4).

As we review below, many NIBS protocols can lead to persistent effects on cortical excitability reflecting synaptic mechanisms of long term potentiation (LTP) or depression (LTD). This feature has promoted therapeutic applications in neurological and neuropsychiatric disorders.

In addition, NIBS techniques, have been used as memory and in general as cognitive enhancers.

However, despite the great therapeutic potential of NIBS apart from major depression where TMS has received formal approval from FDA, there is little consensus for a therapeutic use in other neurological and neuropsychiatric conditions (5–7). The reason of this failure is that we need to improve our knowledge on the mechanisms of action of NIBS in order to overcome the variability across individuals (8, 9).

In the present review, will discuss on the mechanisms underlying the neuroplastic effects of NIBS to consider the possible strategies to improve therapeutic effects.

## NIBS: Available Techniques

### Repetitive Transcranial Magnetic Stimulation

In addition to probing motor cortex excitability with single pulses, TMS can also produce long-term changes in excitability if the TMS pulses are applied repetitively (10).

As a rule, at a regular frequency, low-frequency (1 Hz or less) rTMS reduces cortical excitability (11), whereas high-frequency (5 Hz or greater) rTMS boosts up cortical excitability (11, 12).

This is quite simplistic view, as recent findings have shown that that continuous 5 Hz rTMS decreases instead of increasing corticospinal excitability (13).

rTMS can be delivered in complex burst pattern such as thetaburst stimulation and quadripulse stimulation (QPS) that produce a more reliable effect than conventional rTMS (14–16).

Continuous TBS (cTBS), delivered for 20 or 40 s, decreases cortical excitability, while intermittent TBS (iTBS; applied for 3 min) facilitates cortical excitability. QPS with a short interstimulus interval (e.g., 5 ms) between the 4 pulses facilitates MEPs, while longer interstimulus intervals (e.g., 50 ms) suppress MEPs (16).

Paired associative stimulation (PAS) exploits the principles of Hebbian plasticity imported from animal studies.

PAS was first developed by using low frequency repeated pairing of electrical stimulation of the median nerve combined with TMS over contralateral M1.

Timing is crucial as corticospinal excitability is augmented when the interval between the afferent stimulus and TMS is equal or slightly longer than the individual latency of the cortical N20 component of the median nerve somatosensory-evoked potential. On the contrary, PAS reduces excitability when the interval is shorter than the N20 latency (17, 18).

PAS can also be applied at high frequency (5 Hz), in this case the induction protocol is quite short (2 min) and the intensity of the TMS is under motor threshold. In this way, it is possible to obtain pure cortical effects without effects on spinal excitability (19).

Finally, several variants of PAS protocol have been reported in literature where TMS pulse over the primary motor cortex is paired by another input delivered in remote interconnected cortical areas and even sub-cortical from deep electrodes in patients with deep brain stimulation (20–27).

### Transcranial Electrical Stimulation

The techniques widely used in literature are tDCS, transcranial alternating current (tACS), and randon noise stimulation (tRNS) (28).

In tDCS a tiny electrical current (1–2 mA) is delivered over the skull via 2 soaked sponge electrodes. This small amount of current can polarize neurons by changing their firing frequency (28).

Anodal stimulation induces a cortical facilitation whereas cathodal stimulation over the motor area causes suppression (28).

tACS has opened a new window for stimulating the brain at a predetermined frequency with potential therapeutic applications.

It was first developed in animal models applied where tACS can modulate the phase and frequency of discharges in the brain slice models (29). tACS has been used in humans to entrain cortical rhythms via a frequency- specific empowerment as well as to frequency-specific phase realignment of endogenous brain oscillations (30).

Finally, tRNS is another possible way of modulating cortical excitability using a low intensity biphasic alternating current where the frequency varies continuously in a random manner between 0.1 and 640 Hz (full spectrum) or 101–640 (high spectrum) (31).

## NIBS and Neuroplasticity: Basic Mechanisms

The after effects induced by NIBS are short lasting (~30–120 min) in comparison with the long-lasting effects induced in animal model that last for hours to days (32).

Nevertheless, the effect induced with NIBS are more reminiscent of the labile early phase of LTP/LTD (33). In addition, it is likely that other mechanisms are involved such as post-tetanic potentiation (PSP) and short term potentiation (STP) (34).

### Transcranial Magnetic Stimulation

Despite TMS-induced plasticity shares certain properties described in animal models, however this assumption must be taken with caution since more direct proofs of physiological mechanisms provided by animal studies are still lacking.

On the other hand, pharmacological manipulation of TMS-induced after effects have shown some features reminiscent of long term potentiation (LTP) and depression (LTD) described in animal work.

Indeed, it has been reported that dextromethorphan and memantine, glutamatergic antagonist may prevent TMS after effects (35).

Post-synaptic calcium plays a pivotal role in determine whether a glutamatergic synapse is potentiated, depressed, or left unchanged (36).

The role of calcium in TMS induced plasticity has been investigated in different protocols, for instance plasticity induced by PAS and cTBS300 are modulated differently by different drugs acting on voltage-gated Ca2+-channels (37).

As outlined above, although NIBS-induced plasticity shares some properties reminiscent of NMDA glutamatergic plasticity, this assumption should be taken with caution. Nevertheless, *in vitro* studies lead on organotypic preparations have shown that theta rTMS may interact with glutamatergic neurotramsmission even with a structural remodeling of dendritic spines (38, 39).

In addition, rTMS reduces GABAergic strength at dendritic synapses which could represent a permissive factor for inducing subsequent LTP phenomena (38).

Where these synaptic changes do take place at a system level? Epidural recordings do suggest that for instance PAS affects later I-waves, which reflect the activity located not in the cortico-spinal neurons but on the dendritic tree of an excitatory interneuron involved in I3 wave generation (40). For instance, PAS after effects are abolished if later I-waves of the TMS pulse are suppressed by applying a subthreshold conditioning pulse during the protocol (41).

Although the TMS protocols seem to interact with neural plasticity mechanisms we cannot immediately assert that they have a therapeutic role unless we can demonstrate that these artificial paradigms interact with natural behaviors in a useful way. Several studies suggest that this is indeed the case.

TMS-induced changes in motor cortical plasticity interact with learning of simple motor tasks according the rules of metaplasticity.

Metaplasticity is a term used in basic neuroscience describing how synaptic plasticity can be influenced by the previous synaptic history (42). In keeping with the principles of metaplasticity a motor task can change the amount of a subsequent PAS protocol. Indeed, the amount of a facilitatory PAS 25 ms was reduced after a motor task while the after effects of inhibitory PAS 10 ms was increased (18).

Similar effects, reminiscent of metaplasticity, were also described in QPS (16) and TBS (43).

### Transcranial Electrical Stimulation

As outlined above tDCS is the most popular technique in clinical practice while tACS and tRNS are more used in a research context (28).

tDCS affects cortical excitability in a polarity-specific manner, anodal stimulation over M1 depolarizes neurons increasing cortical excitability while cathodal hyperpolarizes neurons inducing the opposite effect (3).

However, this vision is too simplistic as duration, strength and direction of the effects also depend on the duration, polarity, and intensity of tDCS. Indeed, a duration of the stimulus above 20 min can reverse the after effect (44, 45).

It is interesting to note that tDCS can modulate the excitability of cortical areas outside M1 such as visual and somatosensory cortices (46, 47).

The mechanisms of action of tACS is still elusive, it has been suggested that it polarizes neurons in a frequency domain through a mechanism named stochastic resonance (48) inducing lasting effects through spike-timing-dependent plasticity (49).

In contrast to transcranial direct current stimulation (tDCS), after effects of tRNS seem to be not NMDA receptor dependent and can be suppressed by benzodiazepines suggesting that tDCS and tRNS depend upon different mechanisms (50).

## Current NIBS Therapeutic Applications

A group of European experts was commissioned to establish guidelines on the therapeutic use of rTMS from evidence published up until March 2014, regarding pain, movement disorders, stroke, amyotrophic lateral sclerosis, multiple sclerosis, epilepsy, consciousness disorders, tinnitus, depression, anxiety disorders, obsessive-compulsive disorder, schizophrenia, craving/addiction, and conversion (51).

However, there are only 2 conditions where there is a sufficient body of evidence to accept with level A (definite efficacy) the analgesic effect of high-frequency (HF) rTMS of the M1 contralateral to the pain and the antidepressant effect of HF-rTMS of the left dorsolateral pre-frontal cortex (DLPFC) (52).

A Level B recommendation (probable efficacy) is proposed for the antidepressant effect of low-frequency (LF) rTMS of the right DLPFC, HF-rTMS of the left DLPFC for the negative symptoms of schizophrenia, and LF-rTMS of contralesional M1 in chronic motor stroke (53).

Nevertheless, the optimization of stimulation parameters in routine clinical practice in the real world remain to be established.

A level C recommendation (possible efficacy) has been proposed for several conditions:

- LF rTMS of the left TPC on tinnitus and auditory hallucinations;
- HF rTMS (5–25 Hz) of bilateral (multiple) M1 areas on motor symptoms of PD;
- CRPS type I (HF rTMS of M1 contralateral to pain side);
- hemispatial neglect (cTBS of the contralesional left posterior parietal cortex);
- epilepsy (LF rTMS of the epileptic focus), post-traumatic stress disorder (PTSD) (HF rTMS of the right DLPFC);
- cigarette consumption (HF rTMS of the left DLPFC).

In addition, rTMS of DLPFC can be used to empower the effects of antidepressant medication.

Which are the intrinsic mechanisms that permit rTMS and more in general NIBS, to achieve a therapeutic result?

There are two current theories: the “repair model” and the “interactive model.”

The first model posits that NIBS may transiently reshape the dysfunction caused by the disease.

Therefore, NIBS, to be effective, should produce persistent changes in brain circuitry. However, at present there is no evidence that this can happen.

The interaction model proposes that rTMS can help the brain restore itself. Within this framework rTMS, or NIBS more in general, may promote or enhance natural adaptations to injury or chronic disease.

Indeed, the plastic effects produced by NIBS, in the offline stimulation mode, may interact with brain network boosting or reducing plasticity phenomena.

Unfortunately, the main limitation is that many of these studies have been conducted on small scale and have only been performed at a single center, being difficult to evaluate.

The only clinical entity where NIBS is widely recognized as treatment is depression, where large clinical trials have been conducted.

The initial clinical trials of rTMS began more than 20 years ago with investigations in patients with drug-resistant depression (54, 55).

The idea of using rTMS was driven by functional imaging evidence showing that patients with depression have reduced activity in the left pre-frontal cortex (56, 57).

Therefore, the strategy was to enhance the activity of pre-frontal cortex with high-frequency stimulation and to prolong the after effects by applying rTMS during several daily sessions.

Regarding pain, several review and meta-analyses (58–63) suggest that high frequency stimulation of M1 contralateral to the pain side can reduce pain (pain relief >30% in 46–62% of patients and >50% pain relief in 29%).

The efficacy of a single HF rTMS session tends to persist for a few days and may be enhanced and prolonged with session repetition, while optimal stimulation parameters need to be better defined. In addition, the role of rTMS in the therapeutic armamentarium against neuropathic pain remains to be established. On the other hand, it has been shown that HF rTMS of M1could predict the outcome of epidural motor cortex stimulation (EMCS) (64–68). However, rTMS tests can be used only to confirm the indication of EMCS therapy but not to exclude patients from implantation (56, 58).

In keeping with the interaction model (see above), rTMS acts as a relatively non-specific input promoting synaptic plasticity during physical therapy sessions.

There are 3 post-stroke disorders which may benefit cortical stimulation techniques: motor deficit, aphasia and hemineglect.

The strategy is to increase the excitability of the ipsilesional hemisphere or to decrease the excitability of the contralesional hemisphere, which results in a reduction of its inhibitory influence onto the lesioned hemisphere that can promote recovery.

However, it is important to note that this might be a simplistic interpretation of the effects of these protocols since the contralesional hemisphere may play in some patients an adaptive role promoting recovery (see below).

A meta-analysis of the literature shows that an increase in excitability produced by HF rTMS of ipsilesional M1 or a decrease in excitability induced by LF rTMS of contralesional M1 tends to improve motor abilities in stroke patients (Levels B or C recommendations).

On the other hand, there are several unsolved clinical issues. First the therapeutic value of either modality of stimulation remains to be determined with respect to the phase of stroke recovery (acute or sub-acute vs. chronic). Second, the real impact of rTMS in daily practice is still unknown. In addition, there are safety concerns regarding the possible risk of seizure increasing cortical excitability at the site of injury.

On the other hand, the systematic use of LF rTMS to reduce the hyperactivity of the contralesional hemisphere must be considered with caution, because the hyperactivity of healthy hemisphere may be sometimes adaptive and this may promote stroke recovery (69–71).

Therefore, the feasibility of rTMS in long term stroke rehabilitation remains to be determined and we are still far from a daily practice use of rTMS.

A key limitation is perhaps the use of generic, unvarying methodology given the heterogeneity that is characteristic of stroke (72). Therefore, the future in the use of NIBS in stroke would be to better understand the pathophysiological mechanisms and stratify patients for tailored or personalized cortical stimulation therapies (73).

Another application of TMS as therapeutic tool in neurology is on migraine. It has been reported that early treatment of migraine with aura by single pulse TMS resulted in increased freedom from pain at 2 h compared with sham stimulation, and absence of pain was sustained 24 h and 48 h after treatment (74). These results have been confirmed by another subsequent study (75). Based on these findings the US Food and Drug Administration (FDA) in 2017, approved a device capable of delivering a single pulse TMS to relieve pain caused by migraine headaches that are preceded by an aura—a visual, sensory or motor disturbance immediately preceding the onset of a migraine attack.

Finally, rTMS can be used also in pediatric neurology with promising results in different clinical situations such as autism spectrum disorders, attention-deficit/hyperactivity disorder, epilepsy, and cerebral palsy (76).

Yet, most clinical TMS and tDCS studies have been published on adult populations, and extensive research into the clinical utility of TMS and tDCS in pediatrics remains an unmet needed.

Indeed, further research is required to investigate the effects of age-related differences in basic neurologic mechanisms on the safety and efficacy of brain stimulation in the pediatric brain.

In the next session we will discuss the limitations of currently available NIBS techniques (69, 77).

## Variability of NIBS-induced Effects

NIBS after-effects are quite fragile and variable both within and between subjects and this can potentially flaw therapeutic applications. This variability is probably the result of several factors.

Such inter- and intra-individual variability will severely hamper the clinical use of NIBS as a potential treatment of neurological or psychiatric disorders, therefore the underlying reasons for these variabilities need urgently to be explored (70).

### Effect of Voluntary Contraction

TBS after effects are abolished by the contraction of the target muscle, interestingly muscle contraction after cTBS shifts the depression into facilitation while the facilitatory effects of iTBS are enhanced (71).

The nature of the contraction (i.e., tonic vs. phasic) can also influence the after-effects of TBS (76).

Tonic contraction can also influence tDCS reversing the effects of anodal and cathodal stimulation (78) while tonic activation immediately after tDCS abolishes all after effects (79).

Similar effects of voluntary contractions where observed in QPS, where rhythmic hand opening-closing at 1 Hz for 1 min abolishes any effect on corticospinal excitability (80).

The vulnerability of NIBS effects by muscular pre-contraction may be explained by metaplasticity (see above) (81).

### Inter and Intra-Subject Variability

Several studies lead in large cohort of healthy subjects have shown a considerable inter- and intra-individual variability in response to all NIBS protocols.

It has been reported that only half of the subjects tested can be considered as responder to TBS protocols (82). Similarly, Wiethoff and associates reported that only ~50% of subjects could be considered as responders (83).

The response rate of PAS in a large cohort multicentric study lead in Germany was 53% (84).

All together these data suggest that the probability of producing the “expected” response may be lower than 50%, in most NIBS plasticity-inducing protocols.

QPS, a newer form of rTMS, may provide a reduction of variability, with a responder rate ranging from 60 to 80% (85, 86).

In general, the session-to-session, intra-individual variability, is lower than inter-individual variability.

Lopes Alonso and associated reported that about 70% of subjects maintained reproducible responses to anodal tDCS in separate sessions; a similar percentage was reported in another study (9, 87).

Such inter- and intra-individual variability has severely compromised attempts to use NIBS for treatment of neurological or psychiatric disorders. Therefore, future studies are needed to address the reason of such variability to find new strategies to improve NIBS after effects (70).

## Factors Inducing NIBS Variability

Several factors may underlie such a variability and many of them cannot be changed such as age, gender and genetic polymorphisms. Therefore, it is important to control them through the experimental design (88).

Individual brain anatomy can be a potential source of variability especially if we refer to scalp measurements. This can now be corrected by using TMS neuronavigator systems which use individual anatomical brain images to guide the placement of the coil over the region of interest (89).

Another important factor, which is difficult to control, is that the level of ongoing cortical activity interacts with NIBS after effects.

A possible strategy to control neural activity would be to monitor physical activity since it is well-known that it influences NIBS after effects by acting on ion channels. Thus, keeping the subject relaxed could be a way to reduce NIBS variability (90).

Induced cortical activity on purpose may be used to modulate NIBS after effects.

For example, prior muscle pre-contraction may enhance the inhibitory effects of cTBS (91).

This metaplastic effect can be replicated also outside the primary motor cortex; for instance a cognitive task modulating frontal theta wave activity enhanced the antidepressant effect of rTMS (92).

The level of ongoing cortical activity and even its prior history interacts with the effects of NIBS.

The subject attentional focus may profoundly influence NIBS after effects. For instance, PAS after effects are maximized if subject focused on the stimulated hand while the effects are decreased if the subject directed attention on the non-stimulated hand (93).

Menstrual cycle can affect cortical excitability and plasticity; for example rTMS after effects are maximal on day 14 since estradiol reinforces synaptic potentiation by acting on voltage-gated sodium channels (94).

It is also well-known that PAS after effects are lower in the morning in relationship to the circadian rhythms of cortisol and melatonine (95).

Another potential source of variability is represented by genetic factors. It is well-known that subjects carrying the a Val66Met polymorphism in the gene encoding brain-derived neurotrophic factor (BDNF) have a reduced responsivity to NIBS protocols and an altered use dependent plasticity (96). All these factors need to be considered when NIBS is used for therapeutic purposes.

## Is There a Future for Therapeutic NIBS?

The past 20 years have seen the publication of a remarkable number of papers about the potential therapeutic effects of NIBS in conditions ranging from cocaine addiction to stroke and depression.

On the other hand, despite this has stimulated a tremendous amount of research the overall clinical effects are limited except perhaps for depression (5, 51). Therefore, it is necessary to find new strategies to empower the NIBS therapeutic after effects.

### Improving NIBS Variability

In the previous section, we analyzed the possible sources of variability of the NIBS after effects that can be controlled to optimize NIBS protocols.

An important variable that need to controlled is the adaptation of NIBS based on the individual brain anatomy. Advances in physics and computational science will allow to design brain modeling considering the NIBS-induced electrical field, based on bone thickness with local thinning, CSF volume, and gyral folding of the individual brain (97, 98).

For the last 30 years, NIBS techniques, have approached the brain as a black box, ignoring its endogenous excitability at the time of stimulation.

Indeed, there are several evidences pointing out that NIBS effects are state-dependent on a time scale of minutes to hours, depending on the immediate history of neural activity (99) and synaptic plasticity (100). Brain activity changes, on the time scale of seconds to milliseconds, are governed by rhythmic fluctuations in neural excitability within the ascending thalamo-cortical systems and cortico-cortical projections (101, 102). Therefore, NIBS protocols should be optimized not only on neuroimaging data to account for individual differences in functional neuroanatomy but also taking into account the current oscillatory brain state (100).

Perhaps the most promising strategy to enhance NIBS after effects is to monitor neural activity in real time using EEG an then triggering TMS pulses only at pre-defined states (103).

This approach is called state-dependent brain stimulation (BSDBS).

Indeed, the recent advances in combining TMS with EEG have made possible designing stimulation protocols that are controlled by the EEG signal adjusting stimulation in a very direct way, short-circuiting the motor-sensory loop (51).

Although this state dependent approach has a strong theoretical background coming from animal studies, there are only limited evidences that the same synaptic rules can be successfully applied on the human side (104). Nevertheless, EEG brain-state triggered NIBS-in-the-loop set-ups will enable physicians, in the near future, to interfere with their patients' ongoing brain activity with high temporal, spatial and spectral precision.

This approach has several important advantages. Firstly, neuromodulation can be tailored to each patient thus reducing the inter-individual differences in the excitability and connectivity of brain networks (105).

Secondly, monitoring EEG it is possible to detect the time-course of dynamic changes during network reorganization such as during stroke rehabilitation (106).

Thirdly, EEG brain-state triggered NIBS should be recommended since the modifiability of neurons and networks is a function of their recent activity (metaplasticity) and hence this can determine the direction, extent and duration of after effects in neural networks (107).

Finally, another strategy under study is to use the spaced application of multiple NIBS protocols within a session to improve the reliability and extend the duration of NIBS effects.

Traditionally NIBS protocols are delivered or in a single session (once a day) or in multiple sessions (once a day for consecutive days). Hence spaces application could open a new therapeutic window improve the reproducibility of NIBS effects.

There are new evidence suggesting that this spaced approach may be successful.

For instance, the application of two spaced sessions of cTBS over the cortical frontal eye field region increased saccadic eye movement latency for a significantly longer period than a single cTBS protocol (108).

Similarly, spaced stimulation of parietal cortex contralateral to the stroke improves significantly symptoms of visual neglect (109).

Same effects have been reported after the spaced application of tDCS with a significant enhancement of the after effects when the second tDCS application is delivered while the effect of the first tDCS application is still ongoing (110, 111).

Despite these encouraging results of spaced stimulation, the rules governing this new type of stimulation need to be further investigated in future studies.

Indeed, the mere increase in the train duration with several forms of NIBS including TBS (112) and tDCS (45) can reverse the direction of the induced plasticity or abolish the effects (86). At the same time, intensity increase does not necessarily implies an increase in amplitude but may even reverse inhibition into facilitation (44).

Finally, another possible approach is to use pharmacological neuromodulation by varying dopamine, noradrenaline, acetylcholine or serotonin neurotransmitters to empower NIBS induced plasticity.

This is a stimulating new perspective to empower clinical NIBS effects that however have not yet been investigated systematically.

Among the strategies discussed above, spaced application of multiple NIBS protocols is perhaps the most viable tool to empower clinical efficacy of NIBS effects.

However, it is mandatory in future studies, to identify the optimal spacing between stimulation (in the single session) and to run separate studies to improve the after effects using a multisession approach.

Such studies, although time consuming, will be particularly important for improving the so far limited effect sizes of NIBS protocols for treatment of neurological or psychiatric disorders.

Finally, a professionally-supervised protocol for home-based, remotely-supervised tDCS, supported by specially designed equipment and a telemedicine platform, has shown feasibility in research settings. This approach shows promise for reducing patient burden and enabling longer duration of treatment in addition with home telerehabilitation. Indeed, if patients can safely apply tDCS to themselves at home, combining telerehabilitation with tDCS, this approach would be a good opportunity to empower therapy without costly therapeutic face-to-face supervision. For instance, tDCS combined with cognitive training delivered at home induced a better cognitive outcome in comparison with patients who received just the cognitive training alone (113).

This study showed the feasibility of remotely supervised, at-home tDCS and set up a protocol for safe and reliable delivery of tDCS for clinical studies (114).

### Recommendations for Future Clinical Trials

In future studies, special emphasis should be given to improve the quality of clinical trials testing the therapeutic efficacy of NIBS;

Several recommendations should be considered in future studies:

- increase the sample size and use of realistic placebo control and double blinding in keeping with the rules of drug clinical trials;
- use of randomized cross over study designs and advanced statistical methodologies such as cluster analysis.
- improve anatomical targeting by using neuronavigated TMS;
- encourage new research to discover new feasible targets of stimulation;
- increase the dosage of stimulation which is rather low across studies;
- systematic use of priming strategies to empower NIBS after effects;
- defining and improving clinically valid endpoint measures.

## Conclusions

The methodological improvements and the application of the rigid rules of clinical drug trials may hopefully help to reduce the large inter-individual variation in efficacy that currently makes the final clinical outcome rather modest, although the effects may be very pronounced and even long-lasting in individual patients.

Nevertheless, it should be reminded that rTMS could be used as preoperative predictive factor for selecting candidates for surgery and to validate the cortical target of where to implant electrodes for epidural invasive stimulation.

Finally, the use of rTMS should be systematically considered as an add-on treatment in combination with medication, physiotherapy, or psychotherapy, with the aim of improving or accelerating the efficacy of these therapeutic approaches.

This combined strategy, which is currently used in in depression, in combination with antidepressant drugs, and in stroke rehabilitation (with rehabilitation), will possibly boost up processes of cortical plasticity improving and stabilizing the therapeutic effects of rTMS.

## Author Contributions

CT, VR, and AlbC conception and idea of the paper. GC, AleC, and DM critical analysis of the literature. AQ conception and idea of the paper, data interpretation, overall supervision of the review. All authors discussed the results and contributed to the final manuscript.

### Conflict of Interest Statement

The authors declare that the research was conducted in the absence of any commercial or financial relationships that could be construed as a potential conflict of interest.
